# Efficacy of Noofen 250 mg Capsules for the Management of Anxious–Neurotic Symptoms in Patients with Adjustment Disorder

**DOI:** 10.3390/jcm14155570

**Published:** 2025-08-07

**Authors:** Elmārs Tērauds, Guna Dansone, Yulia Troshina

**Affiliations:** 1 Psychiatric Clinic “Pārdaugava” of National Mental Health Centre, 1007 Riga, Latvia; 2 Medical and Clinical Research Department, JSC Olpha, 2114 Olaine, Latvia

**Keywords:** Phenibut, Adjustment Disorder (AjD), ADNM-20, stress-related conditions, International Classification of Diseases (ICD)

## Abstract

**Background:** This study aimed to evaluate the efficacy and safety of Noofen^^®^^ (Phenibut) in patients with Adjustment Disorder (AjD) and to assess the usability of the ADNM-20 (Adjustment Disorder New Module 20-item questionnaire) in routine clinical practice. This is the first study of Noofen^®^ in patients with AjD conducted in Latvia, and it also represents one of the first implementations of the ADNM-20 scale in routine clinical settings, where its applicability has not yet been widely established. **Methods**: A non-interventional observational study was conducted across several general practice offices in Latvia. Patients aged 18–70 with clinical symptoms of AjD, an ADNM-20 total score ≥ 30, and a new prescription for Noofen^®^ 250 mg three times daily for at least three weeks (per routine practice) were included. Exclusion criteria ruled out concomitant psychiatric or severe somatic conditions and use of medications or interventions that could affect AjD symptoms. Patients completed the ADNM-20 before and after treatment, and score changes were evaluated. **Results**: Ninety patients (65 women, 25 men; mean age 48 ± 12 years) completed the study. At baseline, 56.7% had high AjD symptom severity, with work-related stressors most frequently reported as triggers. After three weeks of Noofen^®^ treatment, ADNM-20 total scores decreased significantly (mean reduction 14.8 ± 11.3 points, *p* < 0.001), with greater improvement in core vs. accessory symptoms. Symptom severity shifted, with the proportion of high-severity patients decreasing 2.5-fold, and 14.4% scoring below the AjD diagnostic threshold post-treatment. Noofen^®^ was well tolerated. ADNM-20 showed good sensitivity to symptom change but remained vulnerable to human error during scoring. **Conclusions**: Noofen^®^ significantly reduced AjD symptoms, particularly sleep disturbance, restlessness, and anxiety, and was well tolerated. The ADNM-20 questionnaire proved useful in clinical practice and should be considered for routine use to better recognize and monitor AjD.

## 1. Introduction

### 1.1. Background

Adjustment Disorders are defined as states of subjective distress and emotional disturbance arising in the period of adaptation to a significant life change or a stressful life event [ICD-10], or as a maladaptive reaction or response to identifiable psychosocial stressor(s) [DSM-5, ICD-11].

Adjustment Disorders are reported as the most widely recognized stress-related diagnosis in clinical practice. According to the results of the WPA-WHO Global Survey, which included 4887 psychiatrists from 44 countries and examined the use of diagnostic classification systems in clinical practice, the diagnosis of Adjustment Disorder ranks among the nine most frequently assigned psychiatric diagnoses. It is reported with comparable frequency to Depressive Episode, Schizophrenia, Bipolar Affective Disorder, Mixed Anxiety and Depressive Disorder, Recurrent Depressive Disorder, Generalized Anxiety Disorder, Mental and Behavioral Disorders due to Use of Alcohol, and Specific (Isolated) Phobias [1]. Studies have found a 2% prevalence rate of Adjustment Disorders in the general population and up to 35% in clinical and high-risk samples. However, the population data have been historically limited due to the lack of uniform diagnostic criteria and diagnostic tools [2].

In contrast to ICD-10 and DSM-5 classifications, ICD-11 abandons any subtypes of the disorder and provides a concept with structured diagnostic criteria and a positive symptom catalogue to define the Adjustment Disorder (AjD). Besides AjD, ICD-11 provides a spectrum of stress-associated diagnoses under the codes 6B4x, like post-traumatic stress disorder, complex post-traumatic stress disorder, prolonged grief disorder, reactive attachment disorder, disinhibited social engagement disorder, and other disorders specifically associated with stress. The diagnostic challenges that arise from the symptom similarity, composition, and dynamics across the various conditions and stages of the disease progression lead to different interpretations among medical professionals, and eventually, to impacted medical care and impaired quality of a patient’s life.

### 1.2. Stress and Health

Stress is a common underlying factor for a variety of mental and physical disorders. This diversity calls for a reconsideration of how stress-related conditions are recognized and managed.

Physiologically, stress is characterized as a state of mental tension caused by challenging situations, which is a natural human response that prompts us to tackle life’s challenges and threats [3]. The range of stressors in modern life varies by their nature and duration, triggering disparate responses—immediate and delayed—affecting psychological realms alone, or both psychological and physical aspects.

The stress dynamic can be described in phases, and essentially, the stress response consists of three stages: the alarm reaction stage, the resistance stage, and the exhaustion stage [4]. These phases flow into one another if resolution does not occur.

The initial response to acute stress, known as the alarm reaction stage, occurs in the aftermath of stressful event(s). This phase involves transient emotional, cognitive, and behavioral symptoms, including autonomic signs of anxiety (e.g., tachycardia, sweating, flushing), states of confusion, sadness, anger, despair, overactivity, inactivity, social withdrawal, or stupor. These symptoms typically appear within a few days of the event. In ICD-11, this reaction is classified as acute stress reaction (QE84) under Chapter 24 (Factors influencing health status or contact with health services), and is no longer considered a mental disorder. Once the stressor is removed, symptoms usually resolve.

If stress is not effectively managed, the imbalance continues in the resistance stage, leading to maladaptation in the form of AjD, a condition that typically arises within a month or a few months of the stressor. It is marked by persistent preoccupation with the event or its consequences, increased anxiety, and recurring distressing thoughts, making it difficult to adapt. This results in significant disruptions in personal, social, or occupational functioning. AjD usually resolves within six months unless the stressor persists [5].

If the condition remains unaddressed, it transitions into the exhaustion phase. This stage is characterized by increased physical and mental fatigue and reduced work capacity. Like the previous, the diagnostic criteria and clinical presentation involving fatigue are diffuse and could be related to some diagnostic challenge. This state can be coded under various categories, such as fatigue (MG22) or burnout syndrome (QD85), sharing the same key symptom: fatigue [ICD-11].

A new stress-related diagnosis, exhaustion disorder, was introduced into the Swedish version of ICD-10 in 2005. Since then, use of the diagnosis has increased rapidly. It is a concept combining the entire spectrum of mental and psychological conditions that may arise during the period of exhaustion, highlighting fatigue as the central symptom. Associated symptoms include a decline in mental energy, reduced physical endurance, and longer recovery time after mental exertion. The condition is also associated with insomnia or hypersomnia, memory impairment, and pain sensations [6].

## 2. Materials and Methods

### 2.1. Study Objectives

Having multiple conditions with a similar background, overlapping clinical manifestations, and a lack of verified diagnostic tools, we attempted to analyze the heterogeneity of clinical characteristics in patients diagnosed with AjD using the ICD-11 Adjustment Disorder New Module 20-item questionnaire (ADNM-20) and to evaluate the efficacy and safety of Noofen^®^ (Phenibut) in patients with AjD.

### 2.2. Intervention

Treatment options for AjD include non-pharmacological interventions like psychotherapy and self-intervention programs, as well as pharmacotherapy [7]. Noofen^®^ (Phenibut) (ATC code N06BX22), manufactured by JSC Olpha, Olaine, Latvia is a phenyl derivative of the neurotransmitter γ-aminobutyric acid (GABA). Phenibut possesses both nootropic and anxiolytic (tranquilizing) properties characteristic of GABA derivatives. It reduces anxiety, agitation, and fear, and improves sleep. Phenibut significantly diminishes symptoms of asthenia and vasovegetative disturbances, including headache, feeling of heaviness in the head, sleep disturbances, irritability, emotional lability, and increases mental work capacity, as well as ameliorates psychological characteristics like attention, memory, rate, and accuracy of sensory–motor reactions. Phenibut, as a GABA-B receptor agonist, improves mental well-being and increases interest and initiative, and motivation for activity in patients with asthenia and emotional lability without causing unwanted sedation or agitation [8,9].

Under the brand name Noofen^®^, Phenibut is registered as a prescription medicine in Latvia and several other countries inside and outside the European Union. Authorized indications of Noofen^®^ include asthenic and anxious–neurotic states (restlessness, anxiety, fear) and insomnia—the symptoms represented also in AjD. Therefore, this study aimed to evaluate the efficacy of Noofen^®^ within the frame of AjD. The secondary objective was to test the usability of ICD-11 corresponding to the ADNM-20 (Adjustment Disorder New Module 20-item questionnaire) in general practice in Latvia.

### 2.3. The Adjustment Disorder New Module 20-Item Questionnaire (ADNM-20)

The ADNM-20 questionnaire is a theory-driven self-assessment tool that measures AjD according to the new diagnostic concept of the ICD-11 [10].

Its validity and psychometric properties have been evaluated in studies for diagnostic concept and for sensitivity to symptom change during the treatment of AjD. The questionnaire consists of two parts: a stressor list and an item list. The stressor list captures a broad range of acute and chronic life events. The item list measures symptoms on a 4-point Likert scale from 1 (never) to 4 (often). An item (symptom) list is divided into six subscales: preoccupation (4 items), failure to adapt (4 items), avoidance (4 items), depressive mood (3 items), anxiety (2 items), and impulse disturbance (3 items). Preoccupation and failure are referred to as core symptoms of an AjD diagnosis (AjD-C), the rest as accessory symptoms (AjD-AS). The ADNM-20 distinguishes between people with low, moderate, and high symptomatology. For moderate symptom severity, a cut-off summary score of 47.5 (or AjD-C 19.7) is recommended for use in science and practice. The thresholds for low and high symptom severity are assumed as 30.1 (12.7) and 59.6 (26.4) points, respectively [11].

Validity and sensitivity to change in ADNM scales in patients with AjD have been investigated, demonstrating ADNM symptom decrease during treatment, replicating the patterns of the Hamilton Anxiety Scale, Sheehan Disability Scale, and Clinical Global Impression Scale. These findings indicate that ADNM is a suitable measurement to assess respective symptom severity changes over time and that it agrees with change patterns of other established measures [12].

The original version of the ADNM-20 questionnaire was translated into the local language, undergoing a full linguistic validation process including dual forward- and back-translation, author review, cognitive debriefing, and analysis. To evaluate the symptom change, after the treatment, patients were asked to complete only the second part (symptoms list) of ADNM-20 [10].

### 2.4. Statistical Analysis

The number of subjects was pre-determined in the study protocol without power-based sample size calculation. Up to 100 subjects were planned to be involved, respecting a real-time patient flow and project time frame for the final number. The ADNM-20 scores before and after the three-week treatment course with Noofen 250 mg hard capsules were presented in absolute and relative frequencies. Observed differences were evaluated at 5% significance. A 95% confidence interval was computed using t-distribution. Data analysis was performed using IBM^®^ SPSS Statistics, v29 (IBM Corp., Armonk, NY, USA).

### 2.5. Adverse Events

Adverse drug reactions (ADRs) were recorded and reported as per routine medical practice in line with national pharmacovigilance procedure defining serious ADR as one that results in death, is life-threatening, requires inpatient hospitalization or prolongation of the existing hospitalization, results in persistent or severe disability or incapacity for work, is a congenital anomaly, or is otherwise medically significant, and non-serious ADR as one that does not correspond to any of the criteria for a serious ADR [13]. As per the study protocol, all signs and symptoms experienced by the patients were collected for analysis.

## 3. Results

### 3.1. Characteristics of the Study Population

Ninety-six patients were involved between June 2023 and May 2024. Eligible patients who have taken Noofen 250 mg hard capsules three times a day for three weeks (21 +/− 3 days, at least 60 capsules in total) and who have pre- and post-treatment assessment data available were included in the efficacy analysis. Six patients were excluded from analysis: two patients who were lost to follow-up, two patients reporting low treatment compliance, and two patients reporting concomitant interventions that are not accepted by the study protocol. No patients were excluded due to efficacy or safety concerns.

**Figure 1:**
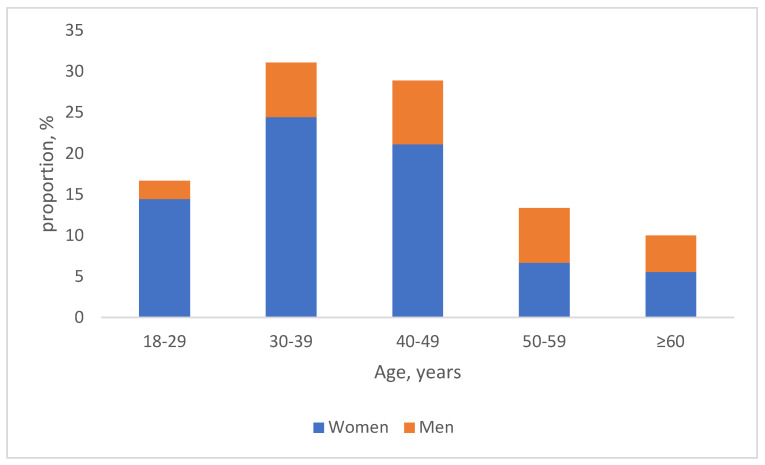
Age and gender structure of the study population (*n* = 90).

The data from 90 patients (65 women and 25 men, 18–69 (48 ± 12) years old) demonstrating clinical symptoms of Adjustment Disorder and having a baseline ADNM-20 total score of at least 30 (34–80 (60.3 ± 10)) points were further analyzed (Figure 1).

Around 85% of the patients indicated the presence of several (2–6) stressors related to AjD, while 9% indicated a single stressor, but 6% indicated up to 10 different stressors. “Too much/too little work” was indicated most commonly (63% of all patients), followed by “Pressure to meet deadlines/time pressure” (47%) and “Family conflicts” (46%), Table 1. The majority of patients had high symptom severity at baseline based on the ADNM-20 summary score (Table 2).

The follow-up assessment was performed within one week after the last day of Noofen^®^ intake in 84% of cases, or later (max within 4 weeks) in other cases. The self-reported symptom severity decreased significantly both for ADNM-20 summary points and for all subscales, leading to a shift in the disposition of symptom severity (Table 2). The rate of patients with high symptom severity decreased by two and a half times, but 14% of the patients went under the lower cut-off value for AjD. Overall, the observed difference was higher for AjD core symptoms compared to accessory symptoms. For the core symptoms, participants exhibited a statistically significant improvement following treatment, with a mean change of 14.8 points (SD = 11.3), highlighting the intervention’s practical significance; the standardized effect size (Cohen’s d) was 1.31 (95% CI 1.0–1.52), which supports the intervention’s strong impact on the observed outcome.

**Figure 2:**
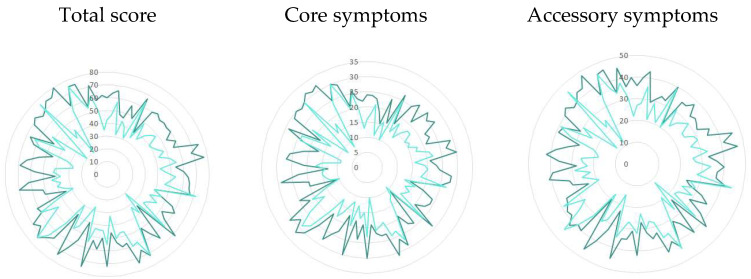
ADNM-20 scores per patient before (outer curve) and after (inner curve) the three-week treatment course (n = 90). A moderate positive correlation for the total score was observed between baseline and post-treatment values (r = 0.5), indicating a trend in which patients with higher baseline symptom levels tend to show greater improvement.

Per patient change in total ADNM-20 score varied from negative numbers (minus 60–1 points, 92.2%) to positive numbers (plus 1–6 points, 3.3%), or no change (0 points, 4.3%), and there was a moderate positive correlation between the baseline and after-treatment scores (r = 0.5), which indicates that patients with more severe symptoms at baseline tended to improve more notably (Figure 2). No correlation was observed with patient age (r = 0.05) or gender.

### 3.2. Change in Individual Items of ADNM-20

**Figure 3:**
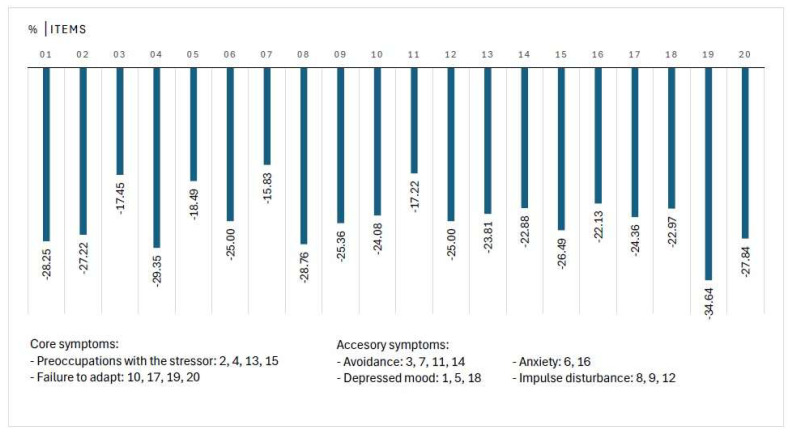
Change in self-reported symptoms for individual items of ADNM-20 questionnaire. The largest difference (35%) was observed for Item 19, which relates to sleep disturbance, followed by Item 4, which addresses overthinking a stressful situation. In contrast, smaller differences were found for items on the avoidance subscale (Items 3, 7, and 11).

The rating for individual items decreased by at least 15% for any of them (Figure 3). The greatest difference (35%) was observed for Item 19, which is related to sleep disturbance (“Since the stressful situation, I can no longer sleep properly”), followed by Item 4 (“I keep having to think about the stressful situation and this is a great burden to me”).

A notable positive dynamic was observed also for the items that are related to authorized indications of Noofen 250 mg hard capsules like restlessness (Item 8: “I am nervous and restless since the stressful situation”) and fear and anxiety (Item 6: “If I think about the stressful situation, I find myself in a real state of anxiety”, Item 16: “Since the stressful situation, I am scared of doing certain things or of getting into certain situations”).

In comparison, less notable changes were observed for Item 7 (“I avoid certain things that might remind me of the stressful situation”) and two other items from the avoidance subscale (Item 3: “I try to avoid talking about the stressful situation wherever possible”, Item 11: “I try to abolish the stressful situation from my memory”).

### 3.3. Consistency Between Patient and Clinician Reported Outcomes

After the treatment course, the clinician provided an assessment of patients’ AjD, either as improved significantly, improved, or improved compared to the baseline (Table 3). In this study, the ClinRO and PRO were consistent, supporting the psychometric properties of the ADNM-20 questionnaire for sensitivity to symptom change during the treatment of AjD.

### 3.4. ADNM-20 Questionnaire, User Experience

The quality of patient-completed ADNM-20 questionnaires was excellent for the symptom list, whereas a significant proportion of the responders (41%) did not identify the most straining event(s) on the stressors list, which can be caused either by the difficulties to select a specific event or by the communication barrier between medical staff who dispensed the questionnaire and respondents. A row of summary scores was recalculated by the investigators due to the mathematical errors on the ADNM-20 paper version.

## 4. Discussion

The ICD-11 conceptualization of Adjustment Disorder (AjD) departs substantially from earlier classifications (ICD-10, DSM-5), eliminating all subtypes and introducing a unified diagnosis defined by two core symptom clusters: preoccupation with the stressor and failure to adapt, both of which must cause significant functional impairment [14]. This shift toward structured, positive diagnostic criteria has enabled the development of more standardized assessment tools—most notably, the Adjustment Disorder New Module (ADNM). The ADNM has been instrumental in operationalizing the ICD-11 definition, despite ongoing debates regarding symptom dimensionality. In our study, the ADNM exhibited strong psychometric properties, including high internal consistency and construct validity, thereby supporting both the ICD-11 diagnostic model and the practical utility of the ADNM in applied settings [15].

More than half of all study participants with Adjustment Disorder had high symptom severity at baseline according to the ADNM-20 scoring system.

Similar to the literature data, job-related stressors were most common among our study participants presenting with AjD.

After a three-week treatment course, the self-reported symptom severity decreased significantly both for ADNM-20 summary points and for all subscales, and the difference was higher for AjD core symptoms compared to accessory symptoms.

The clinician assessment of symptom change from baseline correlated with ADNM score change, similar to the literature data [12].

A substantial proportion of patients did not indicate the most important stressor in the first part of the ADNM-20 questionnaire. This is most likely attributable to the method of administration—specifically, the lack of explanation provided and the acceptance of incomplete responses by the physician. This observation suggests that ADNM-20 may face certain implementation challenges in clinical practice, which should be appropriately addressed. However, this issue impacts only the stressor overview and does not affect the symptom indicators captured in the second part of the questionnaire. Potential scoring errors could be prevented through the use of an automated (electronic) version of the ADNM-20.

In the present study, Noofen^®^ demonstrated a favorable safety profile. This finding is consistent with a systematic review that analyzed all available publications on the safety of Phenibut, including clinical trial reports and individual case studies. The review concludes that, when used at therapeutic doses, Phenibut is generally safe and well tolerated, with only minor adverse effects reported [16].

### Limitations

This job has several limitations. The data were collected during routine clinical practice with restricted opportunity to evaluate potential effects of various cofounding factors, including patients’ health and daily habits. Although the validity and psychometric properties of ADNM-20 have been evaluated for sensitivity to symptom change during the treatment of AjD, the large-scale data supporting general conceptualization are not available. Finally, the long-term outcomes remain out of the scope of this study, especially considering that a stressor can persist for a longer duration of time, potentially transforming AjD into another type of stress-related condition.

## 5. Conclusions

A significant improvement was observed for the AjD symptoms that are related to the clinical indications of Noofen 250 mg hard capsules, like sleep disturbance, restlessness, fear, and anxiety.

Noofen 250 hard capsules were safe and well tolerated in patients with AjD. No subjects were withdrawn from the treatment due to any adverse events or tolerability issues. One subject reported being dizzy for two days without a causality assessment. No other adverse events were reported.

The ADNM-20 questionnaire was generally well perceived by the users, and the obtained results were consistent between patient and clinician reports; however, the scoring is susceptible to human error and should be addressed carefully. The country-specific version of ADNM-20 should be validated for routine use.

It is also important to note that sleep disturbances as a symptom can be a common manifestation during all phases of stress and fatigue. Therefore, the statistically significant reduction in this symptom suggests a potential role for Noofen in the prevention of exhaustion states.

## Figures and Tables

**Table 1:** Disturbing stressors.

Stressful Life Events *	Reporters, %
Too much/too little work	63
Pressure to meet deadlines/time pressure	47
Family conflicts	46
Conflicts in working life	30
Illness of a loved one	30
Financial problems	30
Death of a loved one	19
Termination of an important leisure activity	19
Own serious illness	17
Moving to a new home	13
Divorce/separation	11
Unemployment	11
Conflicts with neighbors	6
Assault	6
Serious accident	4
Adjustment due to retirement	1
Any other stressful event	28

* ADNM-20 stressor list.

**Table 2:** Change in ADNM-20 measures from baseline.

Summary Points	Baseline, Mean (SD)	After Treatment, Mean (SD)	Difference ^
Total score	60.3 (10.5)	45.5 (12.3)	14.8 (11.3)
Core symptoms	24.0 (4.9)	17.4 (5.2)	6.6 (4.9)
Accessory symptoms	36.3 (6.1)	28.1 (7.4)	8.2 (6.9)
**Subscales points**	**Baseline, mean (SD)**	**After treatment, mean (SD)**	**Difference ^**
Preoccupations with stressor	12.5 (2.7)	9.2 (2.8)	3.4 (2.7)
Failure to adapt	11.5 (2.9)	8.3 (2.7)	3.2 (2.7)
Avoidance	12.0 (2.5)	9.8 (2.8)	2.2 (2.8)
Depressed mood	8.8 (2.1)	6.7 (2.0)	2.1 (2.2)
Anxiety	5.8 (1.4)	4.4 (1.4)	1.4 (1.5)
Impulse disturbance	9.7 (2.0)	7.1 (2.3)	2.6 (2.2)
Symptom severity *	**Baseline**	**After treatment**	***p* value**
Low	12.2%	7.8%	<0.19
Moderate	31.1%	55.6%	<0.001
High	56.7%	22.2%	<0.001
Under the lower cut-off value	n/a	14.4%	-

^ *p* < 0.001. * Based on ADNM-20 summary score according to Lorenz et al. [11].

**Table 3:** Clinician versus patient-reported outcomes (ClinRO and PRO).

Clinician’s Assessment of Symptom Change from Baseline	Change in ADNM-20 Total Score; Points; Mean (SD); Min, Max Difference from Baseline; Significance
Improved significantly (n = 33), 36.7%	−8.8 (4.9) −3; −60, *p* < 0.001
Improved (n = 49), 55.4%	−21.3 (10.5), 1; −19, *p* < 0.001
Not improved (n = 8), 8.9%	0.75 (3.1) −3; 6, *p* = 0.26

## Data Availability

The raw data supporting the conclusions of this article will be made available by the authors, without undue reservation.

## Abbreviations

The following abbreviations are used in this manuscript:

ADNM-20	Adjustment Disorder New Module 20-item questionnaire
AjD	Adjustment Disorder
ATC	Anatomical Therapeutic Chemical Classification
DSM-5	Diagnostic and Statistical Manual of Mental Disorders, Fifth Edition
GABA	Gamma-aminobutyric acid
ICD-10	International Classification of Diseases, 10th Revision
ICD-11	International Classification of Diseases, 11th Revision
n/a	Not applicable
PRO	Patient-reported outcome
QE84	ICD-11 code for acute stress reaction
QD85	ICD-11 code for burnout syndrome
SPSS	Statistical Package for the Social Sciences
ClinRO	Clinician-reported outcome
